# Emergence of Influenza A(H7N4) Virus, Cambodia

**DOI:** 10.3201/eid2510.190506

**Published:** 2019-10

**Authors:** Dhanasekaran Vijaykrishna, Yi-Mo Deng, Miguel L. Grau, Matthew Kay, Annika Suttie, Paul F. Horwood, Wantanee Kalpravidh, Filip Claes, Kristina Osbjer, Phillipe Dussart, Ian G. Barr, Erik A. Karlsson

**Affiliations:** Peter Doherty Institute for Infection and Immunity, Melbourne, Victoria, Australia (D. Vijaykrishna, Y.-M. Deng, M. Kay, I.G. Barr);; Monash University, Melbourne (D. Vijaykrishna, M.L. Grau);; Institut Pasteur du Cambodge, Phnom Penh, Cambodia (A. Suttie, P.F. Horwood, P. Dussart, E.A. Karlsson);; James Cook University, Townsville, Queensland, Australia (P.F. Horwood);; Food and Agriculture Organization of the United Nations, Bangkok, Thailand (W. Kalpravidh, F. Claes);; Food and Agriculture Organization of the United Nations, Phnom Penh, Cambodia (K. Osbjer)

**Keywords:** influenza virus, subtype A/H7N4, zoonotic infection, live poultry markets, influenza surveillance, whole genome sequencing, phylogenetics, Cambodia, viruses, H7N4

## Abstract

Active surveillance in high-risk sites in Cambodia has identified multiple low-pathogenicity influenza A(H7) viruses, mainly in ducks. None fall within the A/Anhui/1/2013(H7N9) lineage; however, some A(H7) viruses from 2018 show temporal and phylogenetic similarity to the H7N4 virus that caused a nonfatal infection in Jiangsu Province, China, in December 2017.

Avian influenza virus (AIV) subtype A(H7) is of concern because it has been a leading cause of zoonotic infections over the past 2 decades (*1*). The A/Anhui/1/2013-lineage A(H7N9) viruses, a leading cause of zoonotic infections in Asia since 2013, have not been detected in the Greater Mekong Subregion, but independent H7 lineages, including H7N3, H7N7, and H7Nx, have been detected occasionally in Cambodia since 2009 (*2**–**4*). H7N3 virus was detected from a duck mortality event in Kampong Thom during January 2017 (*2*), and H7N7 virus was detected in a live-bird market (LBM) in Takeo in September 2017 (*4*). Furthermore, highly pathogenic avian influenza (HPAI) A(H5N1) and low-pathogenicity avian influenza (LPAI) A(H9N2) are endemic in Cambodia (*5*); 59 poultry outbreaks of AIV and 56 human HPAI A(H5N1) cases have occurred since 2006. Although the exact ecologic links are unknown, serologic studies suggest that AIVs of multiple subtypes are frequently introduced into poultry in Cambodia, possibly through cross-border trade or through wild birds (*2*,*6*,*7*).

In December 2017, a 68-year-old woman in Jiangsu, China, who had underlying medical conditions was infected by an LPAI influenza A(H7N4) virus, which led to severe pneumonia and intensive care unit admission, but she recovered and left the hospital after 21 days (*8*,*9*). Genetically similar H7N4 viruses were subsequently detected in contact chickens (*9*,*10*) and aquatic poultry in Jiangsu (GISAID, https://www.gisaid.org), substantiating that the infection was zoonotic and raising concerns of endemicity of H7N4 in the region. Because of the antigenic differences between the A/Jiangsu/1/2018-like A(H7N4) virus and other H7 lineages (*10*), including A/Anhui/1/2013(H7N9) lineage, this newly detected H7N4 virus has been proposed as a vaccine candidate for pandemic preparedness (*10*).

Beginning in February 2018, 2 months after the H7N4 case in China, this virus was detected in ducks in Cambodia; the frequency of detection increased in March and April (*4*). Therefore, because of the novelty of the strain and the association with human infection, we sought to understand the genomic diversity of H7 viruses in Cambodia. 

We characterized the whole genomes (for sequencing methods, see Appendix) of 16 viruses collected during 2015–2018 subtyped by reverse transcription PCR (RT-PCR) as having an H7 hemagglutinin (HA) gene or an N4 neuraminidase (NA) gene; we also included viruses for which the HA or NA could not be typed but that were epidemiologically associated with A(H7) viruses (Appendix Table). We obtained samples from poultry swabs collected across multiple LBMs, slaughterhouses, and poultry collection centers in Cambodia; most H7 viruses originated from domestic ducks (*4*).

All AIV samples collected during February–April 2018 in Cambodia (n = 9) (Appendix Table 1, Figure 1) contained >1 segment with high similarity and common evolutionary origins to the Jiangsu H7N4 samples, whereas AIV collected before this period formed other independent lineages derived from wild birds. Seven H7-HA from viruses collected in 2018 in Cambodia (4 H7N4, 1 H7N5, 1 H7Nx, and 1 H7 with mixed N4 and N7 segments) were most closely related to the HA and NA genes of Jiangsu H7N4 isolates; all 6 N4 NA were most closely related to the NA genes of Jiangsu H7N4 isolates (Figure). We also observed close relationships between the Jiangsu and Cambodia isolates in the internal segments polymerase basic protein 2 (PB2), polymerase acidic protein (PA), and nucleoprotein (NP); most viruses carried a common PA gene (Appendix Figure 1). However, none of the H7N4 viruses from Cambodia shared all segments with Jiangsu isolates, indicating continual reassortment with AIV co-circulating in the region.

**Figure:**
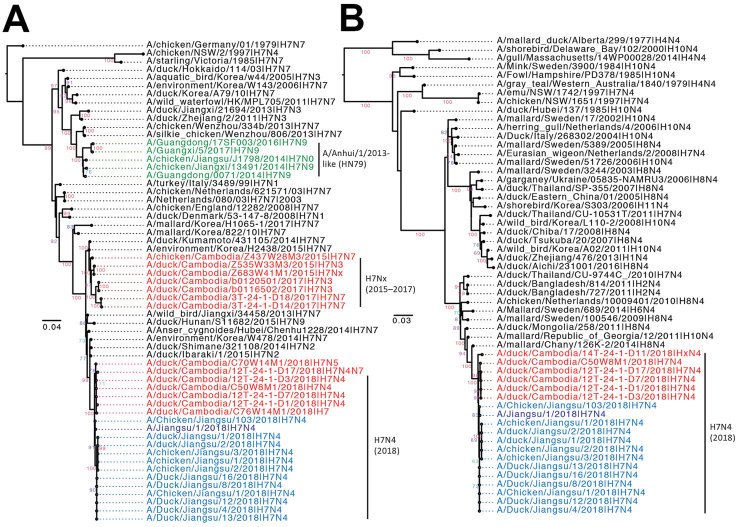
Maximum-likelihood phylogeny of the evolutionary origins of influenza A(H7N4) virus in Cambodia and comparison with reference isolates. H7 hemagglutinin (A) and N4 neuraminidase (B) genes were inferred using a general time-reversible nucleotide substitution model with a gamma distribution of among-site rate variation in RAxML version 8 (https://cme.h-its.org/exelixis/web/software/raxml) and visualized using Figtree version 1.4 (http://tree.bio.ed.ac.uk/software/figtree/). Branch support values were generated using 1,000 bootstrap replicates. Green indicates A/Anhui 1/2013-like lineage viruses; red indicates viruses from Cambodia; blue indicates A/Jiangsu/2018-like viruses. Scale bars represent nucleotide substitutions per site.

Phylogenetic analysis showed that the Cambodia–Jiangsu H7-HA genes emerged during late 2017 (mean time to most recent common ancestor November 2017; 95% CI August 2016–July 2017) and were derived from H7N7 and H7N2 viruses previously detected in aquatic birds in east Asia (Appendix Figure 2). In contrast, the N4-NA exhibited a greater diversity in Cambodia (mean time to most recent common ancestor January 2016; 95% CI January 2015–November 2016) and were derived from H10N4 and H8N4 viruses previously detected in Georgia, Russia, and Mongolia.

Our results show that H7N4 is a newly developing virus lineage that originated from divergent avian lineages within the Eurasian AIV gene pool. The dispersed genetic origins from locations in Europe and central Asia and the similarity of the Cambodia and Jiangsu H7N4 samples indicates that the H7N4 virus was generated in aquatic birds, likely just before their first detection. Detection of H7N4 in LBMs in Cambodia in such a short span of time at such a large spatial distance highlights the risk and potential for rapid spread of AIV lineages throughout the region. The ability to infect a human subject, the continual reassortment and antigenic evolution of this lineage, and the endemicity of numerous LPAI and HPAI viruses may further increase the risk for zoonotic infections and warrants vigilant, active surveillance in wild birds and poultry in the region.

Appendix Additional information about emergence of influenza virus A(H7N4) in Cambodia.
