# Supplementary material for: Emergence of Influenza A(H7N4) Virus, Cambodia
Source: Emerg Infect Dis. 2019 Oct;25(10):1988–91. doi: 10.3201/eid2510.190506 (PMC6759271; doi:10.3201/eid2510.190506)
Supplement: Appendix — Additional information about emergence of influenza virus A(H7N4) in Cambodia. [file 19-0506-Techapp-s1.pdf]

Article DOI: https://doi.org/10.3201/eid2510.190506

Emergence of Influenza A(H7N4) Virus, Cambodia

Appendix

Whole Genome Sequencing

Whole genome sequences were generated by next generation sequencing using Ion Torrent PGM with universal primers, and by Sanger sequencing using gene-specific primers, as described previously (*1*), followed by a customized de novo assembly pipeline of corrected reads using SPAdes v3 (*2*). Consensus sequences were then generated by BLAST against a local database built from all influenza records available in the National Center for Biotechnology Information database (https://www.ncbi.nlm.nih.gov) as of November 2018, followed by maximum likelihood phylogenetic analysis (*3*) using sequences from the GISAID repository (https://www.gisaid.org/; downloaded in January 2019).

Molecular Characterization of Cambodian Influenza A(H7N4) Viruses

None of the Cambodian and Jiangsu A(H7N4) viruses contained known amino acid mutations that confer adaptation of AIV to humans (e.g., PB2 627/701 or HA 186/226/228; H3-HA numbering) (*4*). However, despite showing independent origins, most of the Cambodian viruses contained amino acid mutations in the matrix (M) gene, namely N30D and T215A, shown to increase pathogenicity of A(H5N1) virus in rodents (*5*). All samples contained markers that indicated that these viruses would be sensitive to known antiviral drugs, including adamantanes, oseltamivir/zanamivir and baloxivir-marboxil (*6*).

References

1. Suttie A, Yann S, Y P, Tum S, Deng YM, Hul V, et al. Detection of low pathogenicity influenza A(H7N3) virus during duck mortality event, Cambodia, 2017. Emerg Infect Dis. 2018;24:1103–7. https://doi.org/10.3201/eid2406.172099

2. Bankevich A, Nurk S, Antipov D, Gurevich AA, Dvorkin M, Kulikov AS, et al. SPAdes: a new genome assembly algorithm and its applications to single-cell sequencing. J Comput Biol. 2012;19:455–77. https://doi.org/10.1089/cmb.2012.0021

3. Stamatakis A. RAxML version 8: a tool for phylogenetic analysis and post-analysis of large phylogenies. Bioinformatics. 2014;30:1312–3. https://doi.org/10.1093/bioinformatics/btu033

4. Yamayoshi S, Fukuyama S, Yamada S, Zhao D, Murakami S, Uraki R, et al. Amino acids substitutions in the PB2 protein of H7N9 influenza A viruses are important for virulence in mammalian hosts. Sci Rep. 2015;5:8039. https://doi.org/10.1038/srep08039

5. Fan S, Deng G, Song J, Tian G, Suo Y, Jiang Y, et al. Two amino acid residues in the matrix protein M1 contribute to the virulence difference of H5N1 avian influenza viruses in mice. Virology. 2009;384:28–32. https://doi.org/10.1016/j.virol.2008.11.044

6. Shin WJ, Seong BL. Novel antiviral drug discovery strategies to tackle drug-resistant mutants of influenza virus strains. Expert Opin Drug Discov. 2019;14:153–68. https://doi.org/10.1080/17460441.2019.1560261

7. Suchard MA, Lemey P, Baele G, Ayres DL, Drummond AJ, Rambaut A. Bayesian phylogenetic and phylodynamic data integration using BEAST 1.10. Virus Evol. 2018;4:vey016. https://doi.org/10.1093/ve/vey016

Appendix Table. Low pathogenic avian influenza viruses detected in poultry in Cambodia and their genomic similarity to A/Jiangsu-China/1/2018

Virus name	Subtype	Date	Location	HA†	NA	PB2	PB1	PA	NP	MP	NS
A/duck/Cambodia/12T-24–1-D3/2018	**H7N4‡**	2018 Mar 22	Takeo	**99.4**	**99.4**	**98.1**	96.1	**99.4**	96.7	98.0	97.2
A/duck/Cambodia/12T-24–1-D17/2018	**H7N7/4**	2018 Mar 22	Takeo	**96.7**	**99.4**	95.0	95.7	**99.5**	96.5	N/A	97.2
A/duck/Cambodia/C50W8M1/2018	**H7N4**	2018 Feb 24	Phnom Penh	**99.4**	**99.5**	93.7	96.3	**99.4**	96.5	98.3	97.2
A/duck/Cambodia/14T-24–1-D11/2018	**HxN4**	2018 Apr 10	Takeo	N/A	**99.0**	93.7	95.3	97.4	97.0	97.5	97.8
A/duck/Cambodia/12T-24–1-D1/2018	**H7N4**	2018 Mar 22	Takeo	**99.0**	**99.3**	93.7	95.7	**95.9**§	96.7	97.4	N/A
A/duck/Cambodia/12T-24–1-D7/2018	**H7N4**	2018 Mar 22	Takeo	**99.3**	**99.4**	N/A	N/A	N/A	N/A	N/A	N/A
A/host/Cambodia/C76W14M1/2018	H7Nx	2018 Apr 05	Phnom Penh	**99.2**	N/A	N/A	N/A	N/A	N/A	N/A	N/A
A/duck/Cambodia/C70W14M1/2018	**H7**N5	2018 Apr 05	Phnom Penh	**99.4**	N5	91.9	95.3	95.0	**98.7**	98.2	97.3
A/duck/Cambodia/C8W6M1/2018	H10N7	2018 Feb 06	Phnom Penh	H10	N7	93.5	95.6	94.5	96.5	97.1	97.0
A/duck/Cambodia/3T-24–1-D14/2017	H7N7	2017 Sep 17	Takeo	95.1	N7	91.0	94.4	91.0	97.0	97.4	97.3
A/duck/Cambodia/3T-24–1-D18/2017	H7N7	2017 Sep 17	Takeo	95.2	N7	92.8	94.6	91.7	97.0	97.4	88.8
A/duck/Cambodia/b0120501/2017	H7N3	2017 Jan 12	Outbreak	95.5	N3	86.7	94.9	92.4	88.1	94.7	71.5
A/duck/Cambodia/b0116502/2017	H7N3	2017 Jan 12	Outbreak	95.5	N3	86.7	95.0	92.5	88.2	94.6	71.8
A/chicken/Cambodia/Z437W28M3/2015	H7N7	2015 Jun 09	Takeo	96.7	N7	93.5	96.5	94.9	97.3	98.3	97.9
A/duck/Cambodia/Z535W33M3/2015	H7N3	2015 Aug 13	Takeo	95.9	N3	N/A	N/A	N/A	N/A	N/A	N/A
A/duck/Cambodia/Z683W41M1/2015	H7Nx	2015 Oct 10	Phnom Penh	96.0	N/A	N/A	N/A	N/A	N/A	N/A	N/A

*HA, hemagglutinin; MP, N/A, not available; NA, neuraminidase; NP, nucleoprotein; NS, nonstructural protein; PA, polymerase acidic protein; PB, polymerase basic protein.

†Percentage similarity of strains from Cambodia to corresponding segment of A/Jiangsu/1/2018, with bold values showing similar evolutionary origins. The evolutionary relationships of these segments are shown in the Figure in the main text and Appendix Figures 1 and 2.

‡Bold type represents similarity of surface genes of Cambodian viruses to A/Jiangsu/1/2018.

§Incomplete gene sequence.

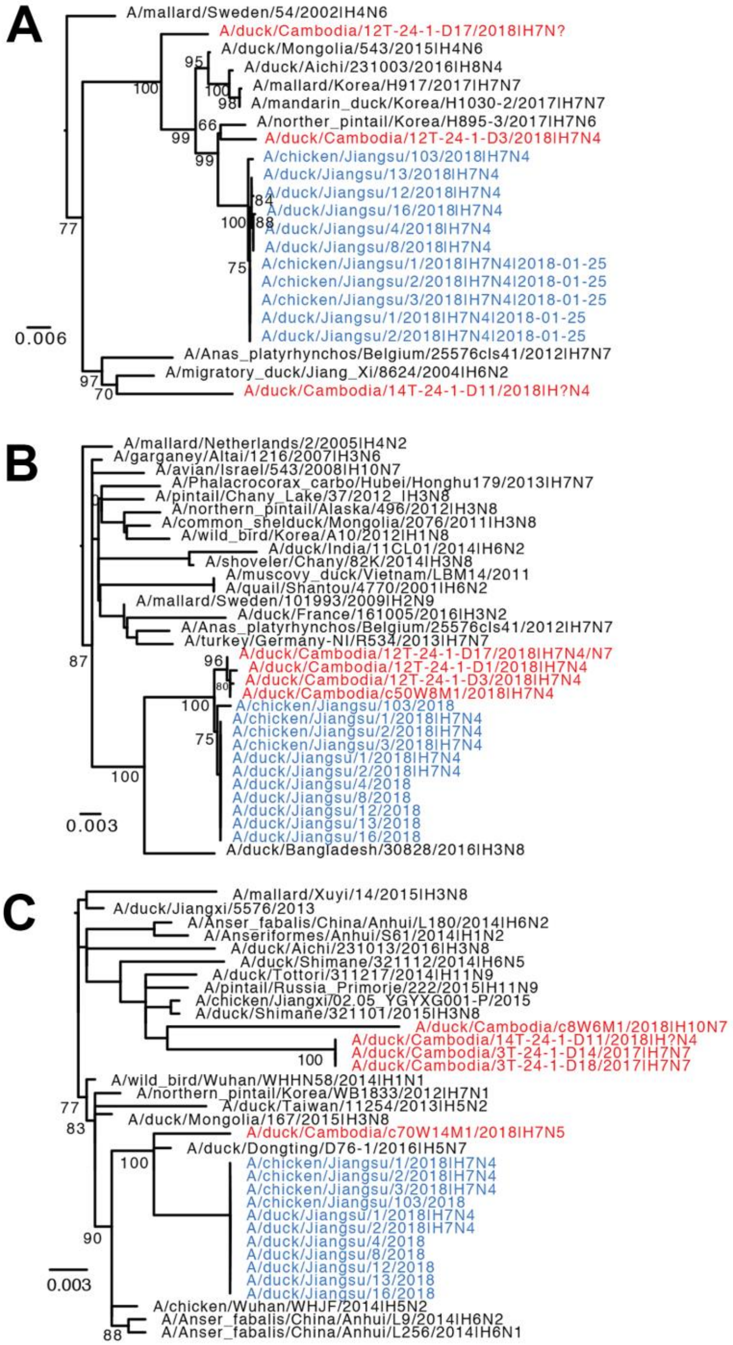

Appendix Figure 1. Maximum likelihood trees of the PB2, PA, and NP gene segments showing the Jiangsu H7N4 and their most closely related strains. Phylogenetic trees were inferred using a general time reversible nucleotide substitution model with a gamma distribution of among-site rate variation in RAxML v8, and visualized using Figtree v1.4 (http://tree.bio.ed.ac.uk/software/figtree). Branch support values were generated using 1,000 bootstrap replicates. Scale bar represents nucleotide substitutions per site.

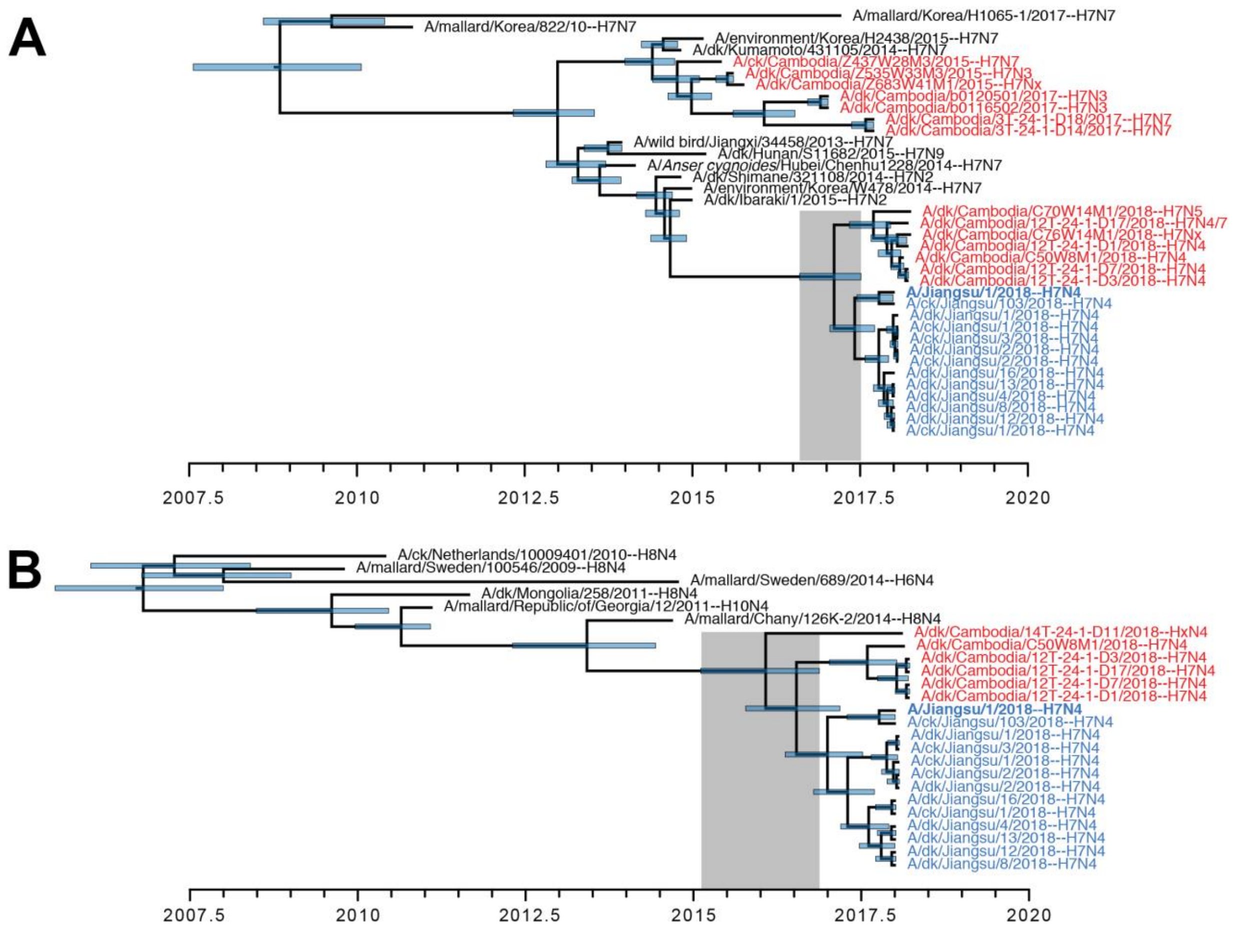

Appendix Figure 2. Time scale of evolution of H7N4 virus. Dated phylogeny of the (A) H7-HA and (B) N4-NA genes inferred using an uncorrelated log-normal relaxed clock model with constant population demographic prior in BEAST 1.10 (*7*), and visualized using Figtree v1.4 (http://tree.bio.ed.ac.uk/software/figtree). Node bars represent 95% confidence intervals.
